# Supplementary material for: Identification and Functional Characterization of CYP4D2 Putatively Associated with β-Cypermethrin Detoxification in Phortica okadai
Source: Genes (Basel). 2022 Dec 11;13(12):2338. doi: 10.3390/genes13122338 (PMC9777750; doi:10.3390/genes13122338)
Supplement: Supplementary file 1 [file genes-13-02338-s001.zip › genes-2052896-supplementary.pdf]

Table S1 PCR amplification primers

Amplification of primers	Gene name	Sequence primers (5′-3′)	Product size(bp)
**Full-length cDNA cloning**	*Pocyp4d2*	F: TCTCGCCCATAAAACGTGAC<br>R: GTAACAAGGAGCTGAAGAAAGG	1448
	Pocyp49a1	F: ATGCCTTCCCTCAAACATTA<br>R: TCACTCGTTGCGTAGTGTTAGT	1224
	Pocyp28d2	F: ATGGCAACAAGCGCAGGC<br>R: GTCGAAAGTGCGCACAATT	900
	Pocyp28d1	F: GCATCAAGACCGCCAAATCG<br>R: ATCAATGCGTTCCGTGTCCT	731
	Pocyp28d3	F: TGGGCCTCATTTATTGCTAT<br>R: GTGATGTCGTTGAGGTGCTAC	370
**Real-time PCR analysis**	*Pocyp4d2*	F: CATTCACACGCACACCCAT<br>R: TCATAACGCCGACATTCCA	189
	Pocyp49a1	F: GTGGTGGCATTCTGCTTTAG<br>R: GTCATATTCCCGCACTTGGT	98
	Pocyp28d2	F: GTCGGTGTGGAGTCCTCTT<br>R: TTTTCACTGTCGGTTTGTTC	158
	β-tublin	F: TCCGCACTTTGAAACTGAC<br>R: CACGAGATGTTAGTGGAGCA	128
**SiRNA synthesis**	*Pocyp4d2*<br>FAM	AUUAUUCUUGGUGAUGUGCdTdT<br>GCACAUCACCAAGAAUdTdT	306

Table S2 Differentially expressed CYP450 genes in *Phortica okadai* with exposed toβ-cypermethrin for 0 h and 1 h

Gene family	Unigene ID	Gene name	C_AVG	T_AVG	Log2（C/T）	up/down
CYP12	DN18226_c0_g1	*cyp12d1*	21.55	5.69	-1.96	down
	DN17809_c0_g1	*cyp12c1*	3.66	1.46	-1.39	down
CYP18	DN15901_c0_g1	*cyp18a1*	6.41	1.45	-2.22	down
CYP28	DN22931_c0_g3	*cyp28d2*	6.46	18.09	1.37	up
	DN22931_c0_g2	*cyp28d1*	15.55	35.50	1.10	up
	DN22931_c0_g1	*cyp28d1*	1.34	7.20	2.33	up
CYP306	DN1606_c0_g1	*cyp306A1*	2.78	0.02	-7.19	down
CYP309	DN19408_c0_g1	*cyp309a1*	12.74	6.78	-1.03	down
CYP315	DN14381_c0_g2	*cyp315A1*	1.14	0.41	-1.57	down
CYP4	DN19194_c0_g1	*cyp4ad1*	1.11	0.18	-2.74	down
	DN20354_c2_g1	*cyp4d2*	1.42	7.88	2.31	up
	DN19325_c0_g1	*cyp4g1*	74.06	8.16	-3.20	down
CYP49	DN17906_c0_g1	*cyp49a1*	0.20	0.94	2.07	up
CYP6	DN16972_c0_g1	*cyp6d4*	2.20	0.88	-1.39	down
	DN16746_c0_g1	*cyp6w1*	63.03	2.30	-4.93	down
	DN18071_c0_g1	*cyp6a22*	24.75	0.51	-5.77	down
	DN22077_c0_g1	*cyp6g1*	19.45	4.40	-2.19	down
CYP450-like protein	DN9970_c0_g2	-	0.36	0.00	-4.35	down
	DN9970_c0_g3	-	0.67	0.00	-6.79	down
	DN2777_c0_g1	-	0.44	0.00	-5.17	down
	DN2777_c0_g2	-	0.51	0.00	-5.55	down

Note: C_AVG and T_AVG means the average expression levels of CYP450 genes in *Phortica okadai* with exposed to β-cypermethrin for 0 h and 1 h

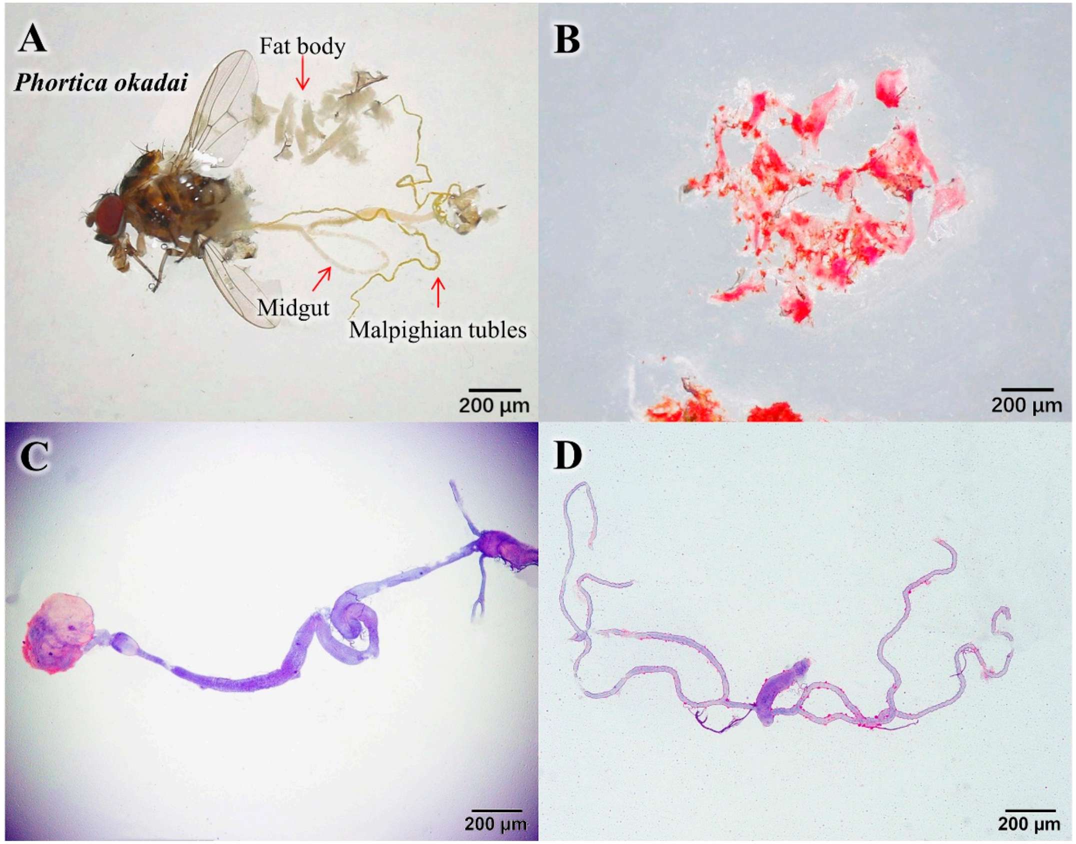

Figure S1 Microanatomy of *Phortica okadai*. (A) Overall view of *Phortica okadai*. (B) Fat body visualized using Oil Red O 0.5% (Solarbio, Beijing, China) staining. (C) Midgut and (D) Malpighian tubes were stained with HE staining kit (Solarbio, Beijing, China) according to the manufacturer's instructions.

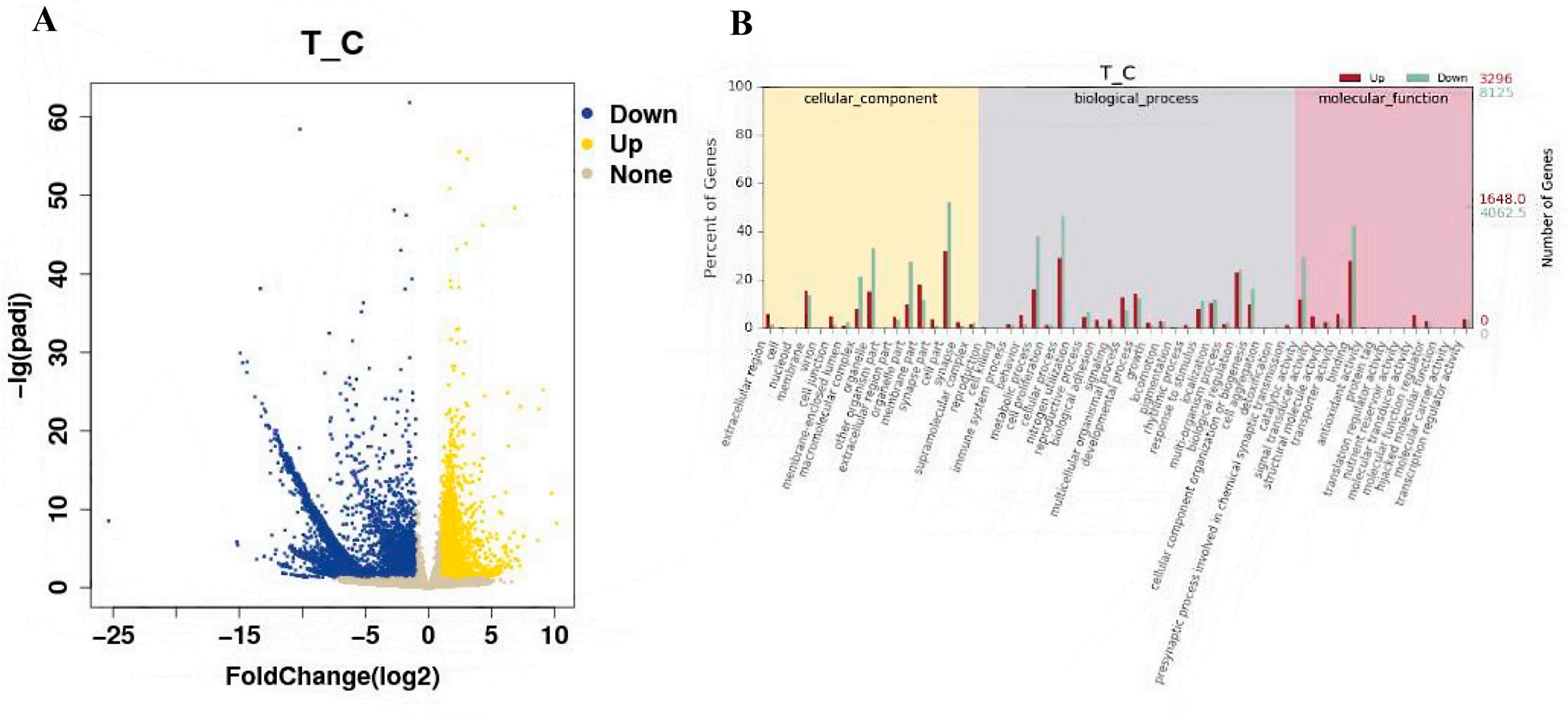

Figure S2 Transcriptome differential expression analysis. (A)Volcano plot analysis of DEGs between 0h (C) and 1h (T) groups. Blue dots indicate DEGs with higher expression in 1 h group, and yellow dots indicate DEGs with higher expression in 0 h group. (B) GO enrichment analysis of DEGs expressed in 0h and 1h groups.

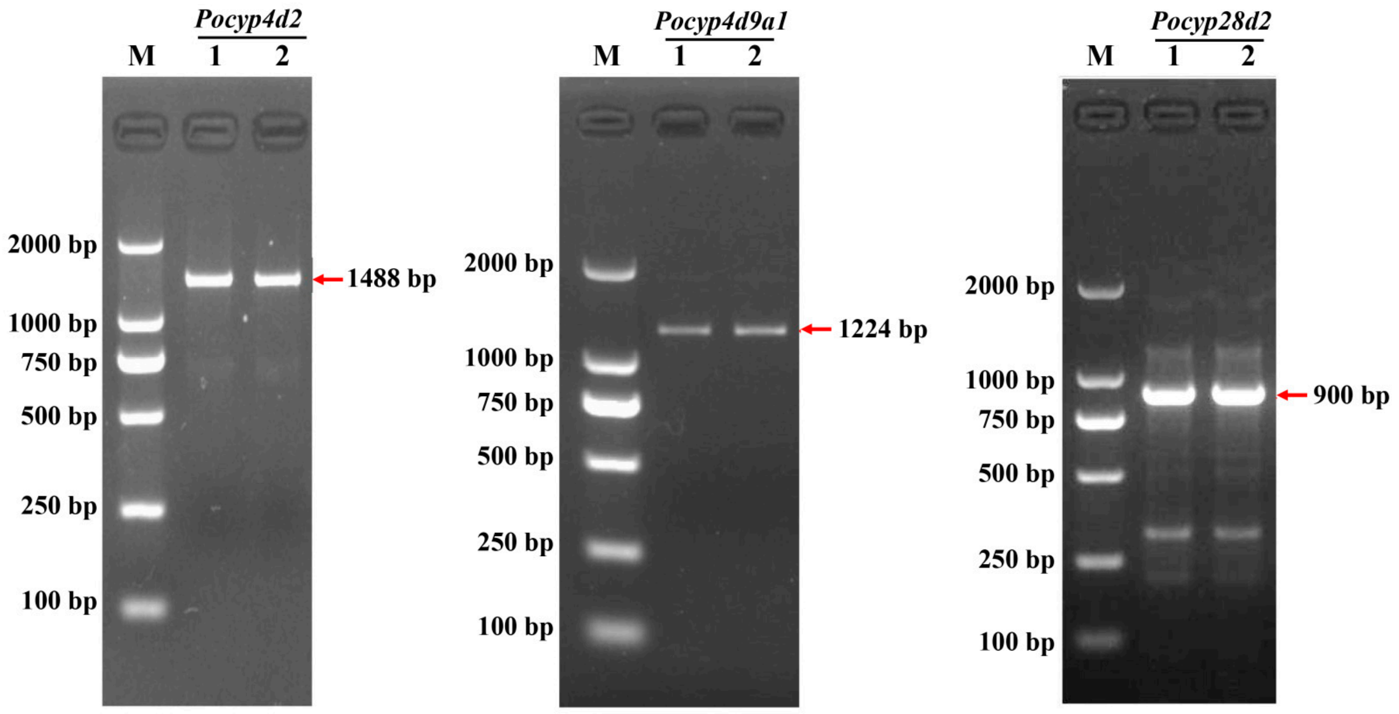

Figure S3 The electrophoretogram of PCR product of the *Phortica okadai* cloned CYP450 genes (M: 2000 bp DNA ladder; lane 1and lane 2: Target gene).

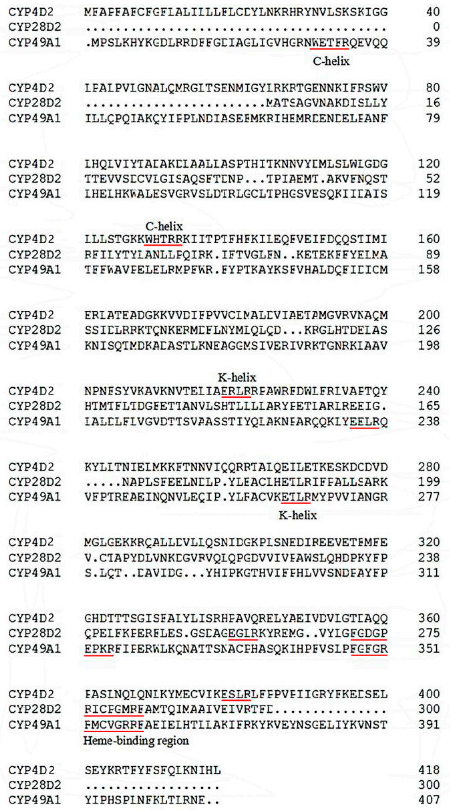

Figure S4 The deduced amino acid sequences of cloned CYP450s from *Phortica okadai* (Red underline indicates the conservative sequence of the P450s)

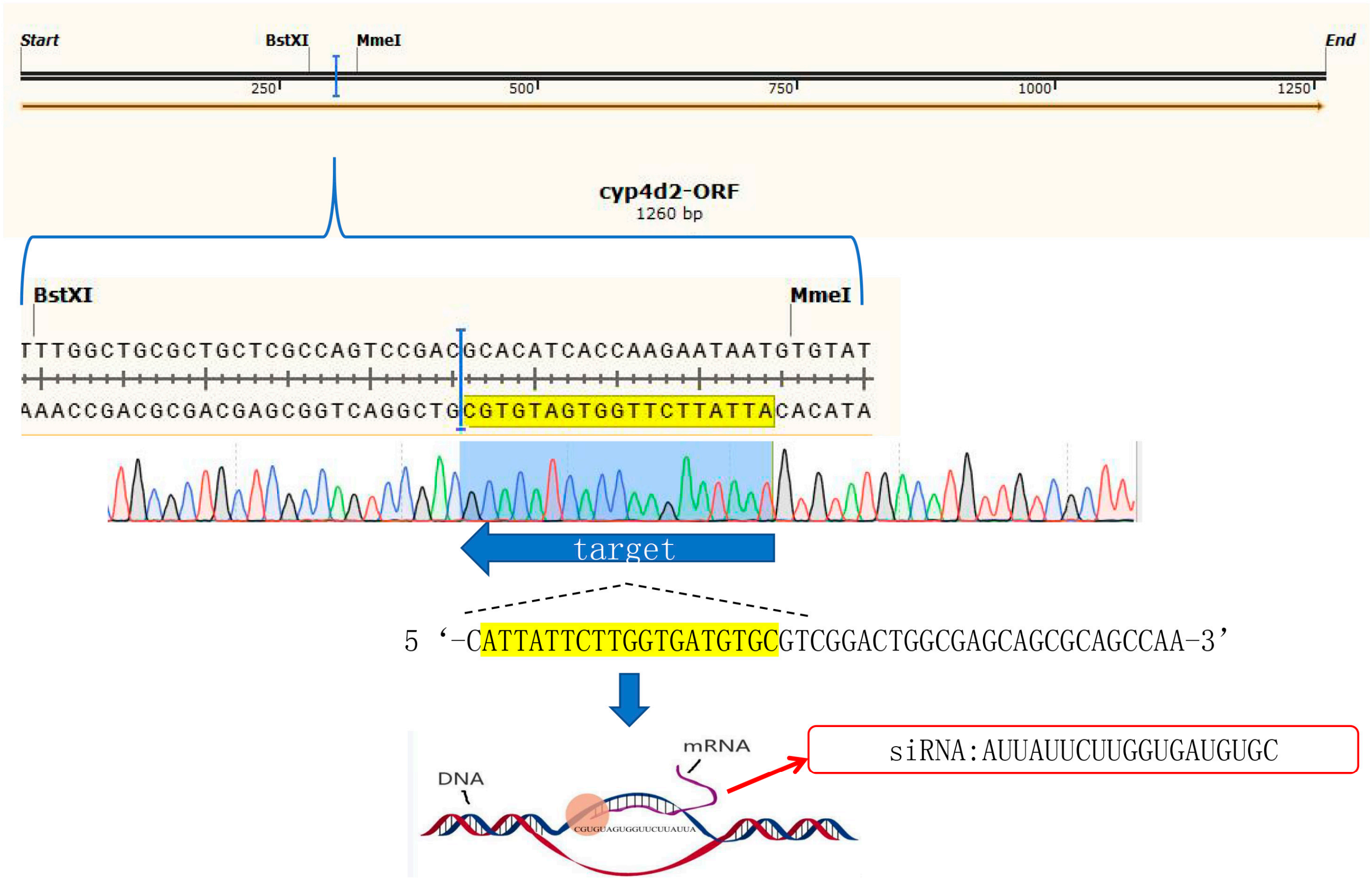

Figure S5 Design map for Pocyp4d2-siRNA fragment in *Phortica okadai*
